# Supplementary material for: Trafficked Malayan pangolins contain viral pathogens of humans
Source: Nat Microbiol. 2022 Aug 2;7(8):1259–69. doi: 10.1038/s41564-022-01181-1 (PMC9352580; doi:10.1038/s41564-022-01181-1)
Supplement: Supplementary file 1 — Supplementary Figs. 1 and 2. [file 41564_2022_1181_MOESM1_ESM.pdf]

nature portfolio

https://doi.org/10.1038/s41564-022-01181-1

Supplementary information

Trafficked Malayan pangolins contain viral pathogens of humans

In the format provided by the authors and unedited

A1, Pangolin copiparvovirus BIME1 (330 bp)

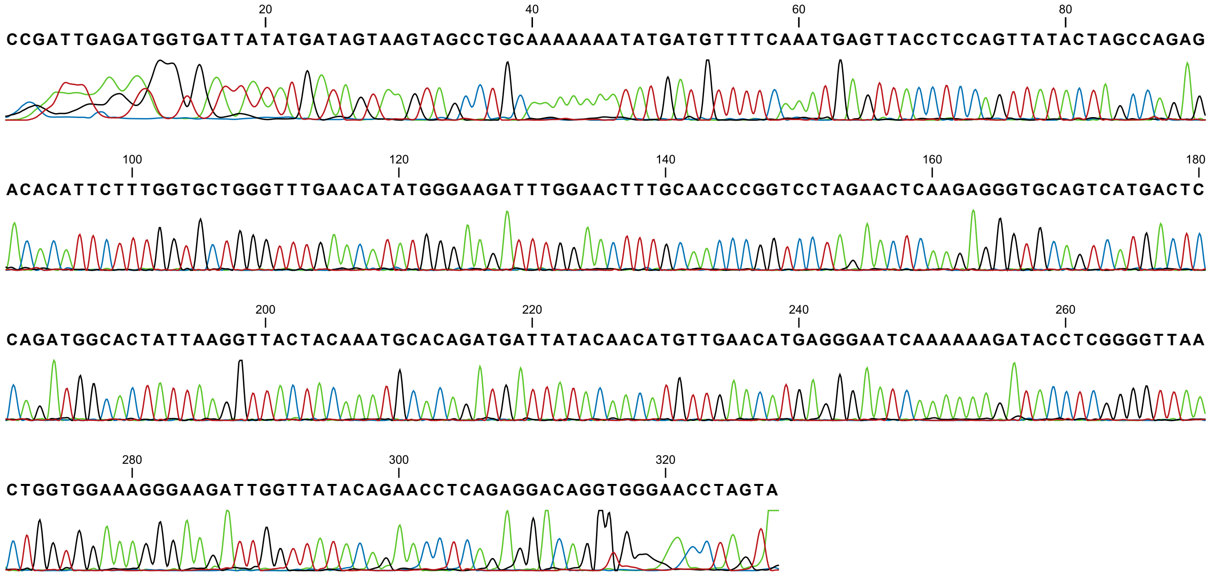

A2, Pangolin RSV-A (450 bp)

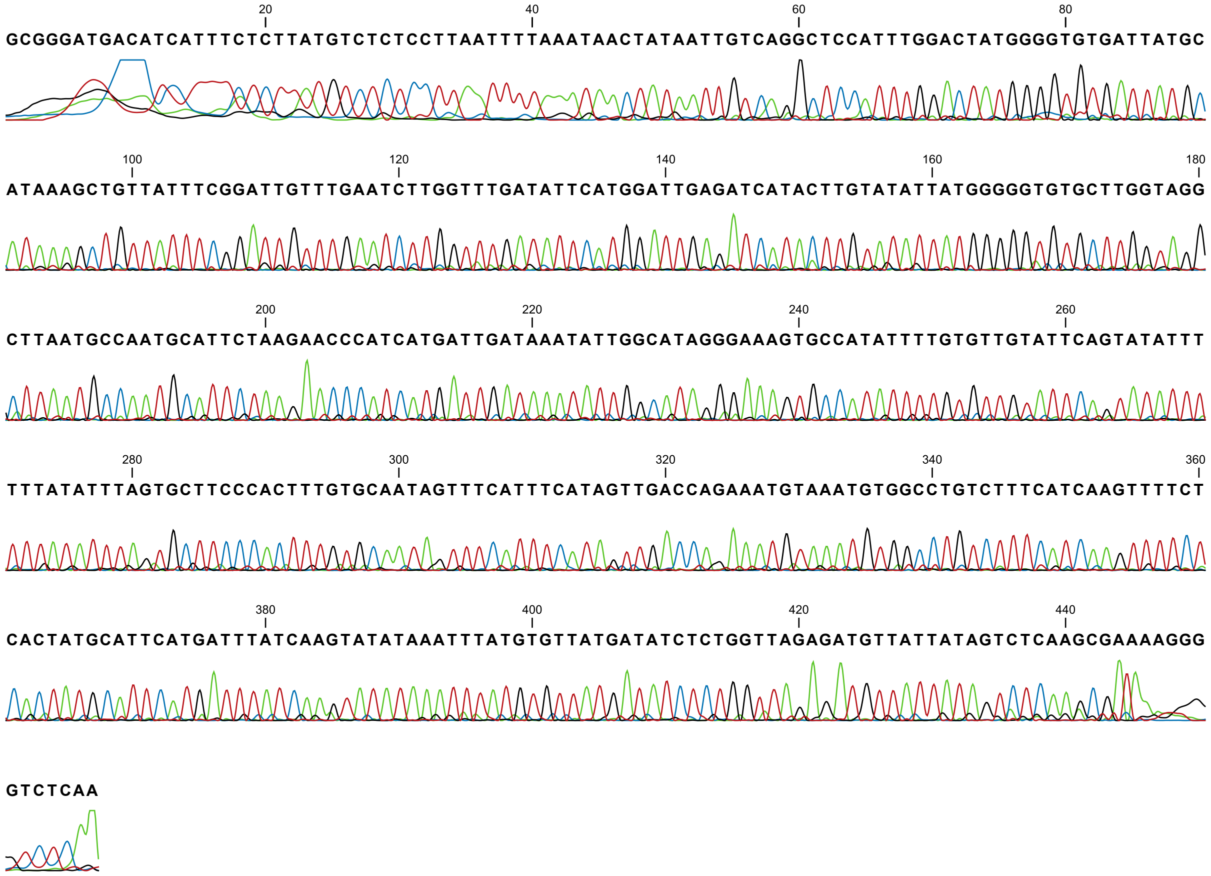

A3, Pangolin RSV-A (450 bp)

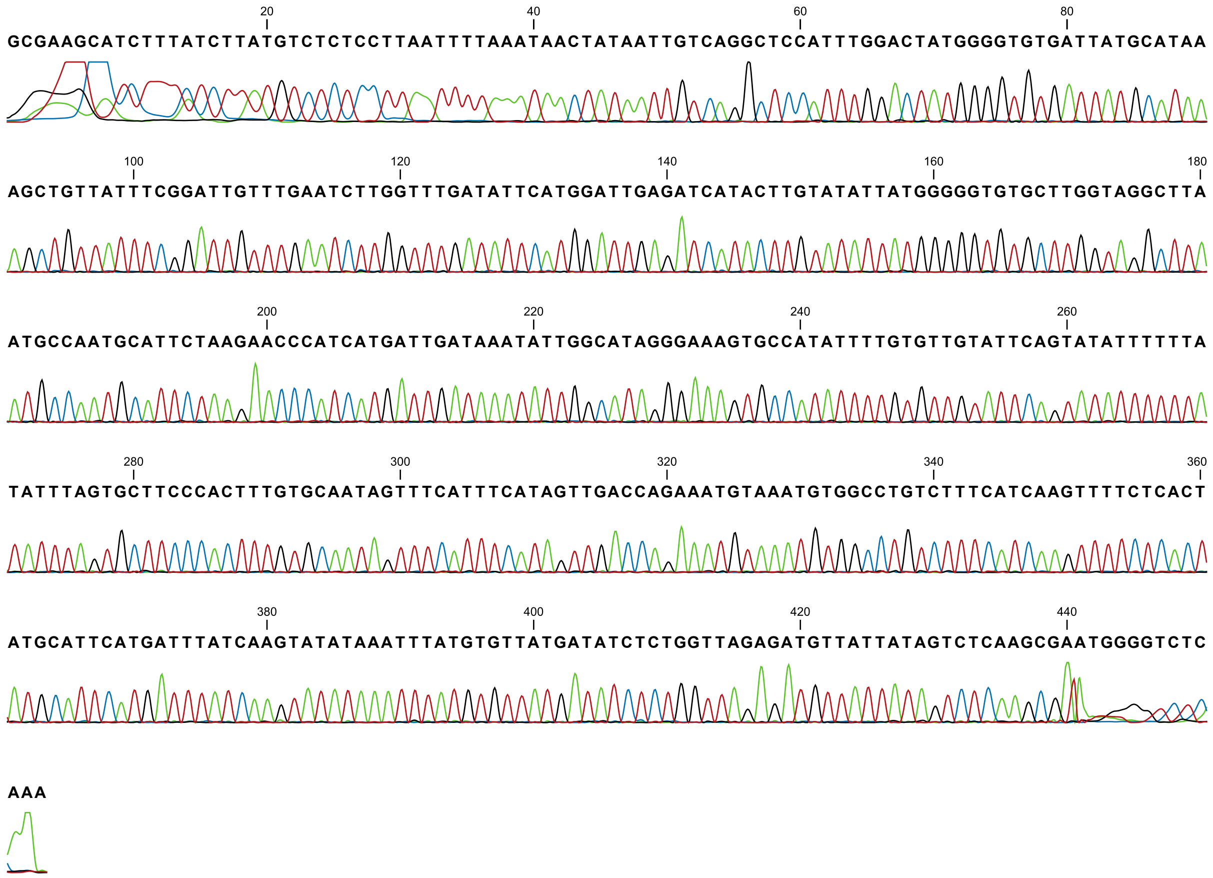

A4, Pangolin hunnivirus BIME4 (481 bp)

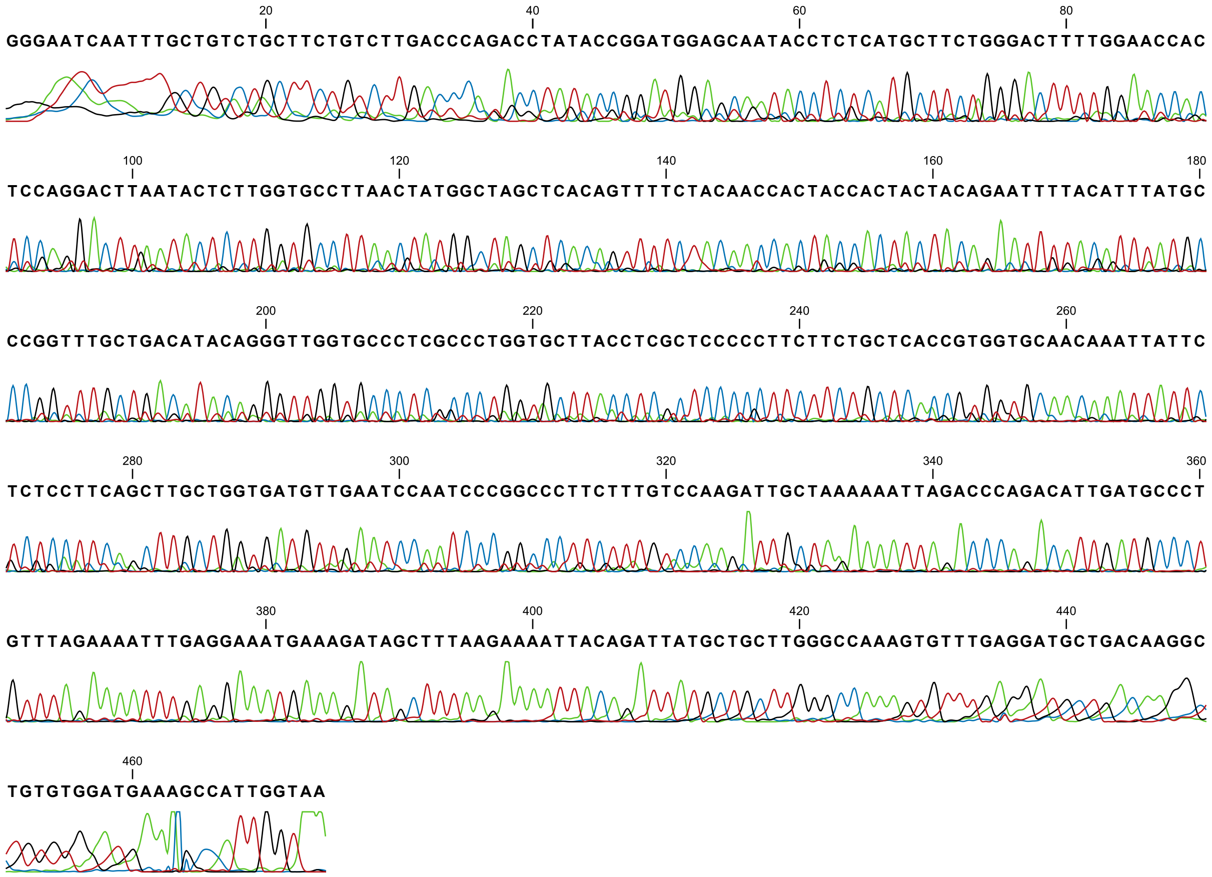

A5, Pangolin hunnivirus BIME1 (467 bp)

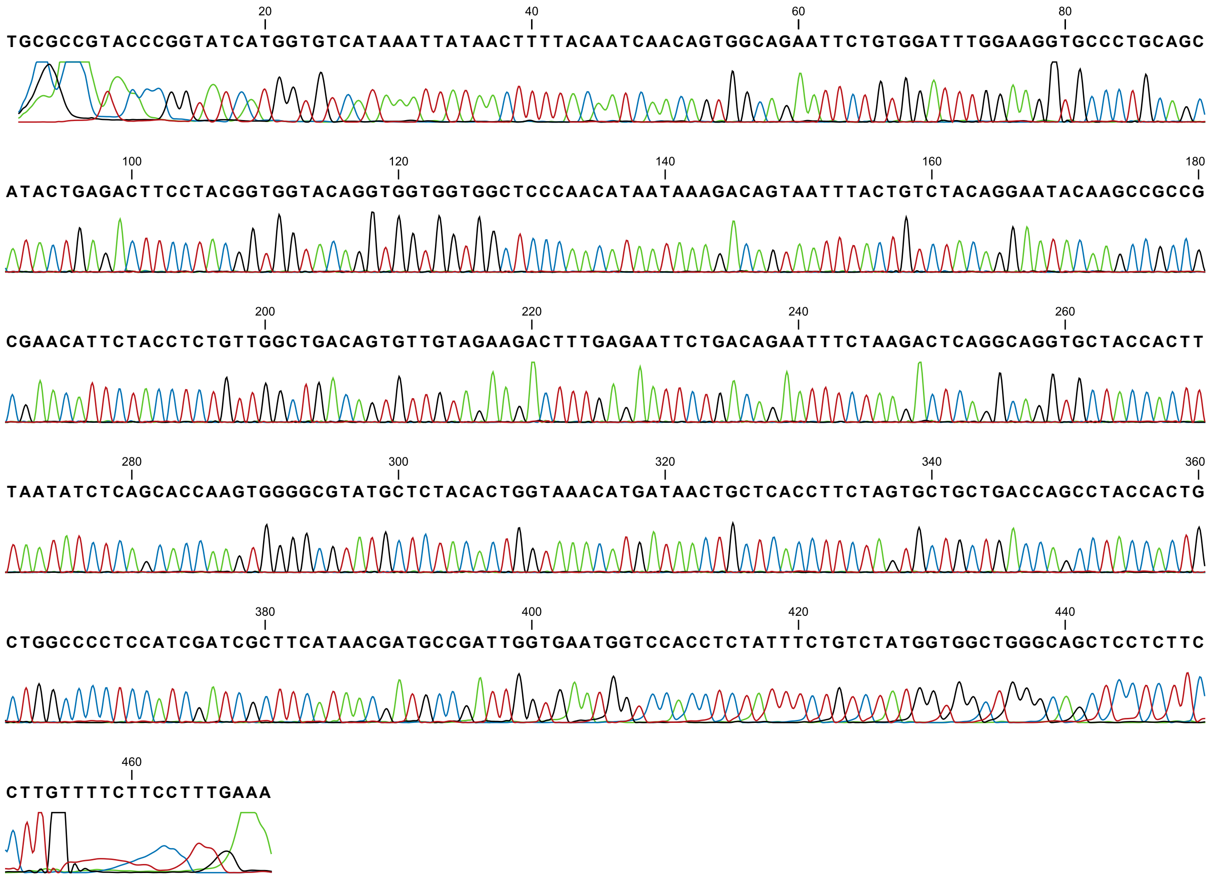

A7, Pangolin hunnivirus BIME5 (410 bp)

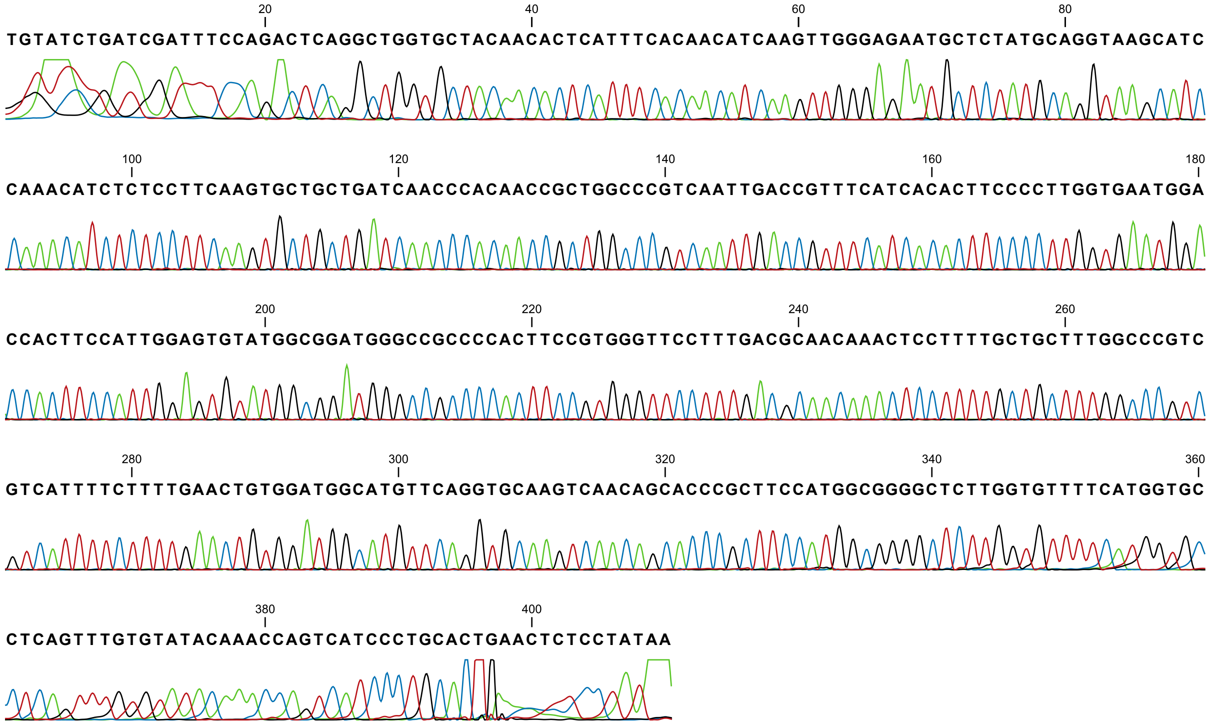

B1, Pangolin pestivirus BIME9 (451 bp)

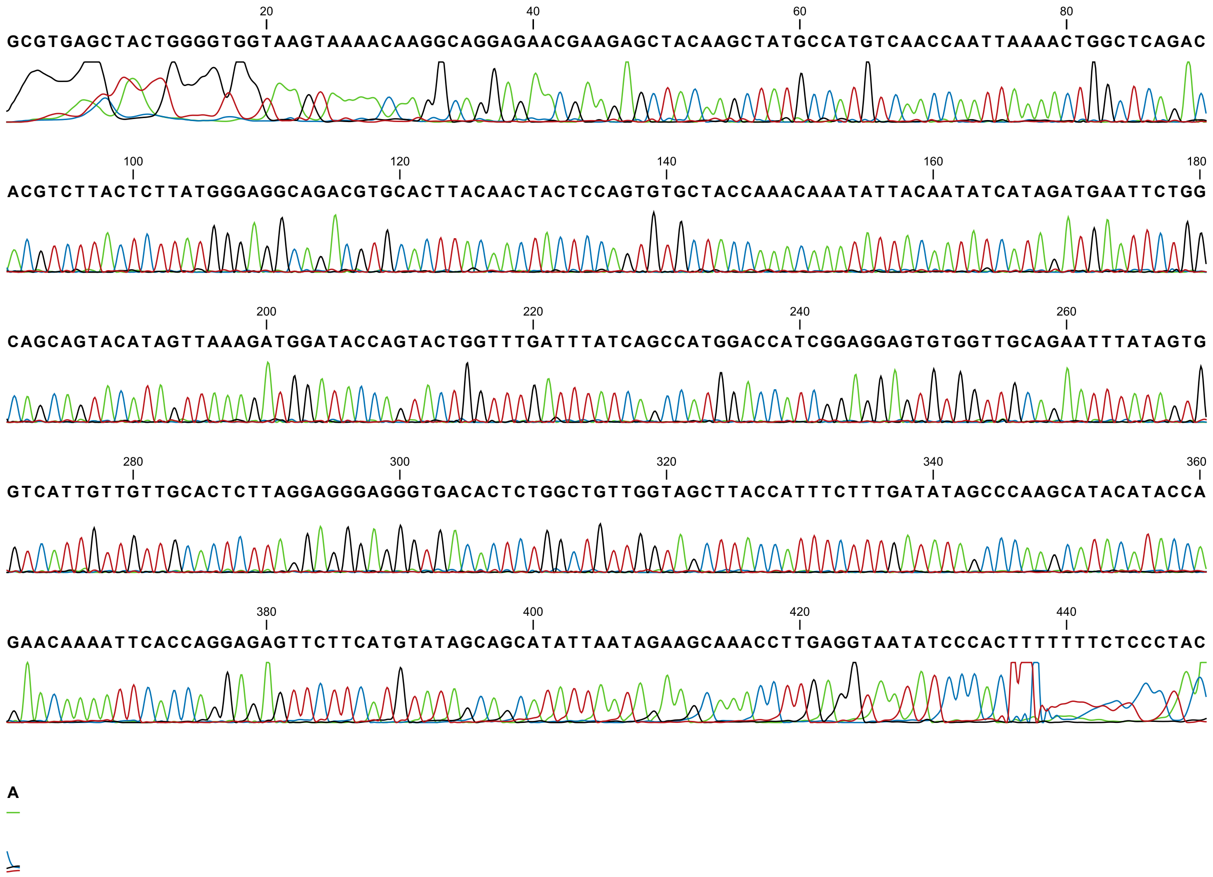

B5, Pangolin pestivirus BIME1 (407 bp)

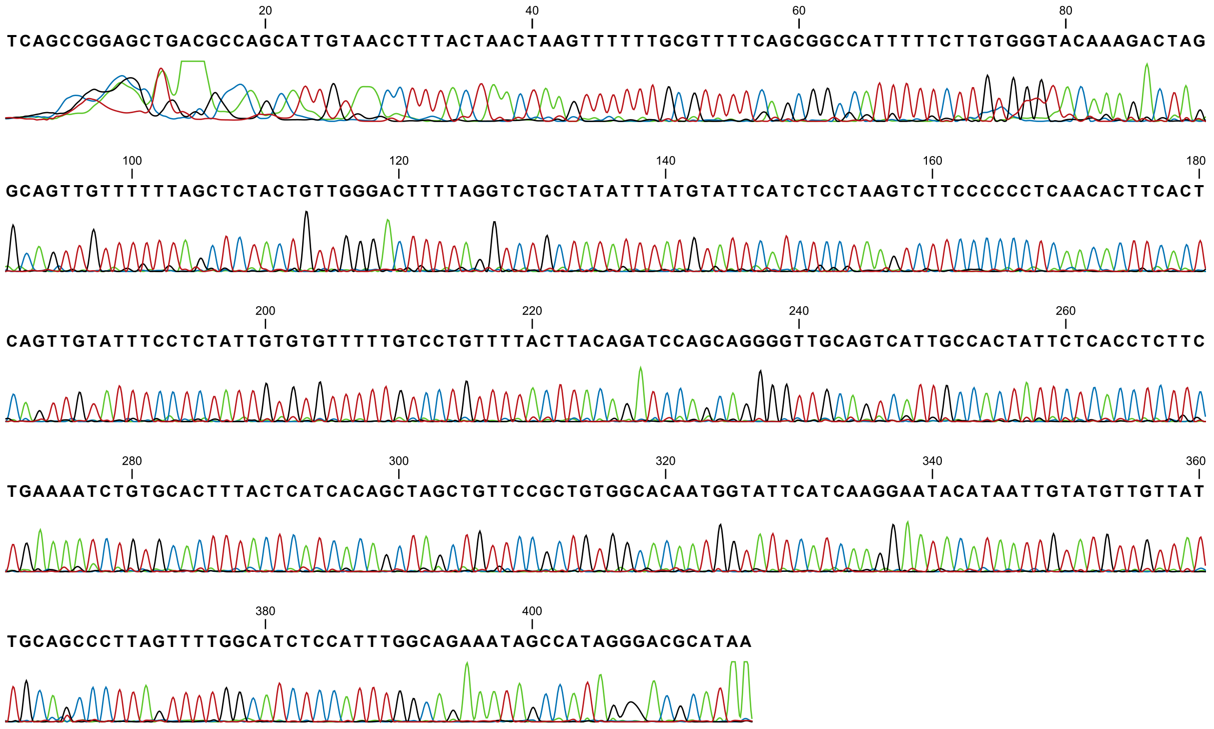

B6, Pangolin pestivirus BIME3 (394 bp)

CCTTAAGCGGCTCCAAGGACTCCGATCAAGTGTGGCATGACCCTGTCAGAATTTGAGGAGAGATTTTATACAAAAATTTTTATAACTGAA

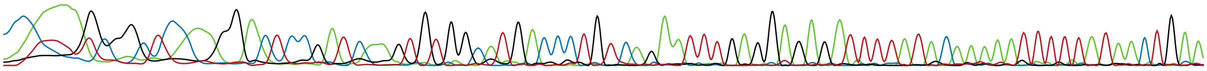

CACAGCCAAAGTGATCATAGATTAGAGGGCCCTGGCAAGATAGTTAGGTATGCAGACAATGGGCTATTGTACCTGAGAAATCTGCCAATA

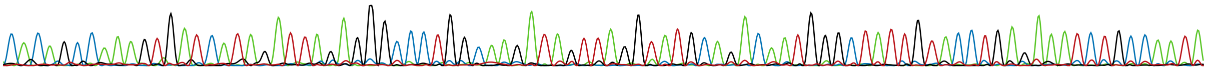

TTGGCGTCTAAACAGAAGATGCTGATAGTGGGCAATCTGGGCAATGAAATCGCAGATCTGGAACATCTCGGGTGGACCCTTAGAGGCCCA

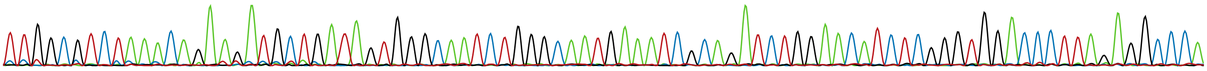

GCCGTTTGCAAAAAAATAACACAAAGCACCAAATGCCAGGCCACAATTATTGACAAAATGACTGCTTTCTTTGGCATAATGCCTAGAGGA

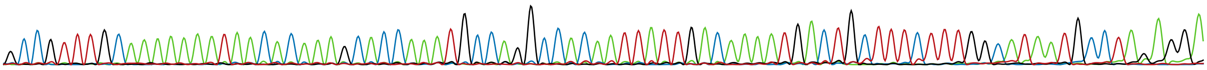

GCTACTCCCAGGGCTCCCACCAGTTCCCCCCTACATAA

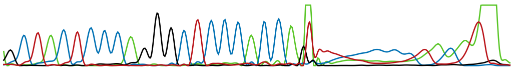

B7, Pangolin pestivirus BIME2 (397 bp)

GCGGACCCTTTGGCGTAGTTGTTTGACCTTGGTGTTACTTGTTGCCCCTAATTCCCTGGCTTGCTCTTTTAAACGTCCAATGTCACCTAT

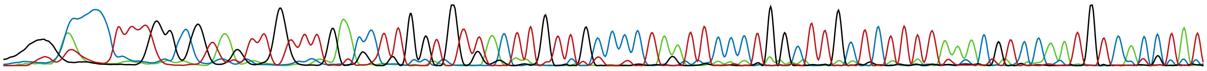

GAAGTGATACTGTTCTCCTTTTAAGCTCAGATCTAAAAATTTTGGCTCGTCATGATGCAGCCATTCTGGCAATTCATAGTGGCTCCCCTC

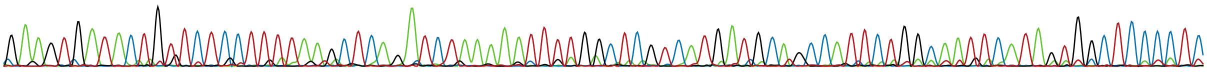

AGTCTTCCCTAGTAACAGTTCTGCTTGTTCTTTGTTTAGCCTTTTACTCTTAGCTTTTCCCCGGCTGAAGGCTTCCAGCAGGTCTCTTGT

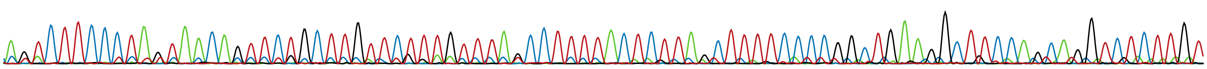

CAAGAAAGGAACTACGTAGTCTGCCAGTCCGTGGAGCGACTCATGTACCTGGTTTCCAACCTGAACAAAAACCACCTGACCTTCAAAGGC

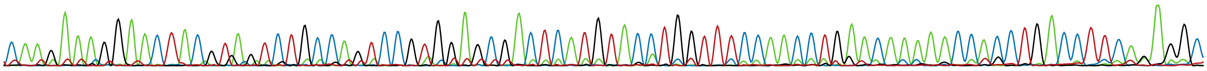

CACCTTCTTTAGGAATCTGAAAGAAGTCTGAAG

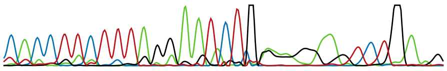

B8, Pangolin pestivirus BIME7 (461 bp)

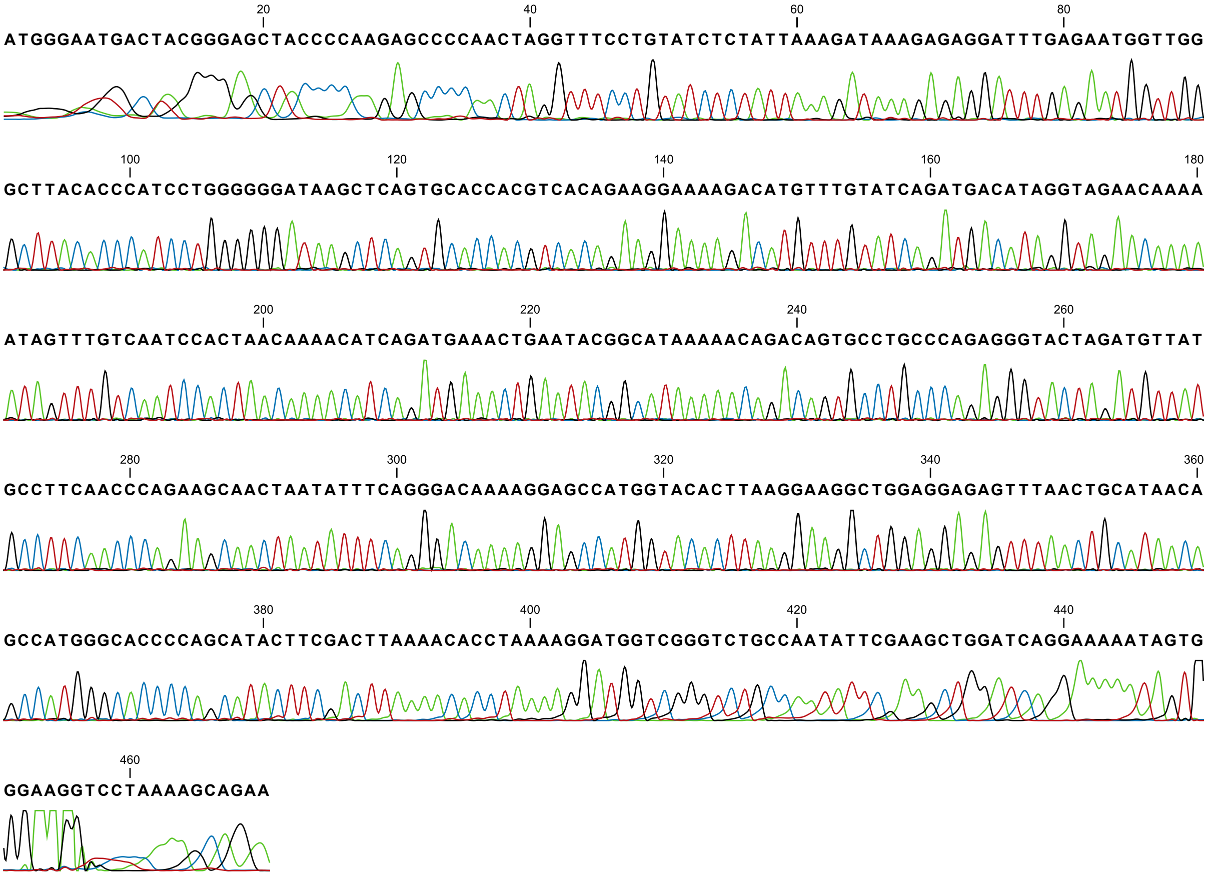

C1, Pangolin pestivirus BIME7 (461 bp)

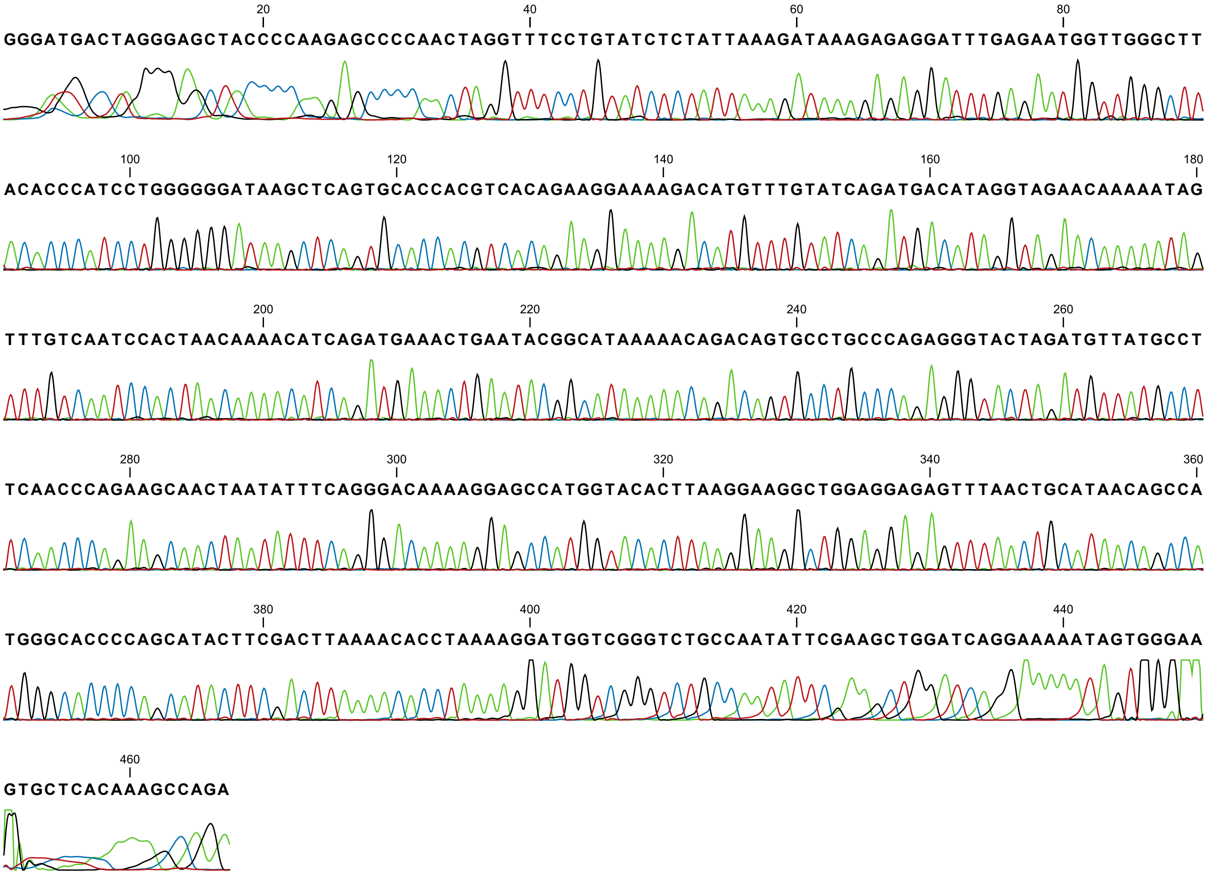

C2, Pangolin pestivirus BIME7 (461 bp)

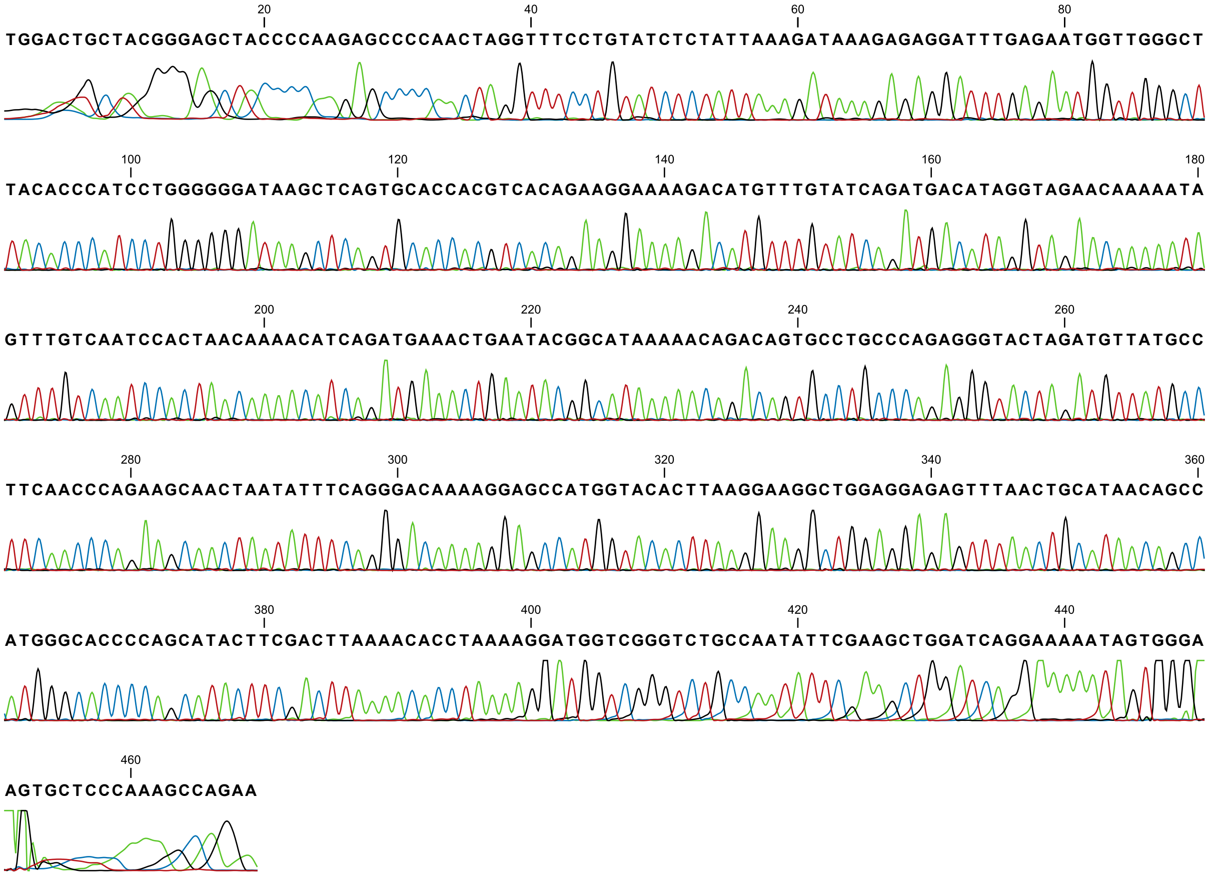

C4, Pangolin phlebovirus BIME1 (402 bp)

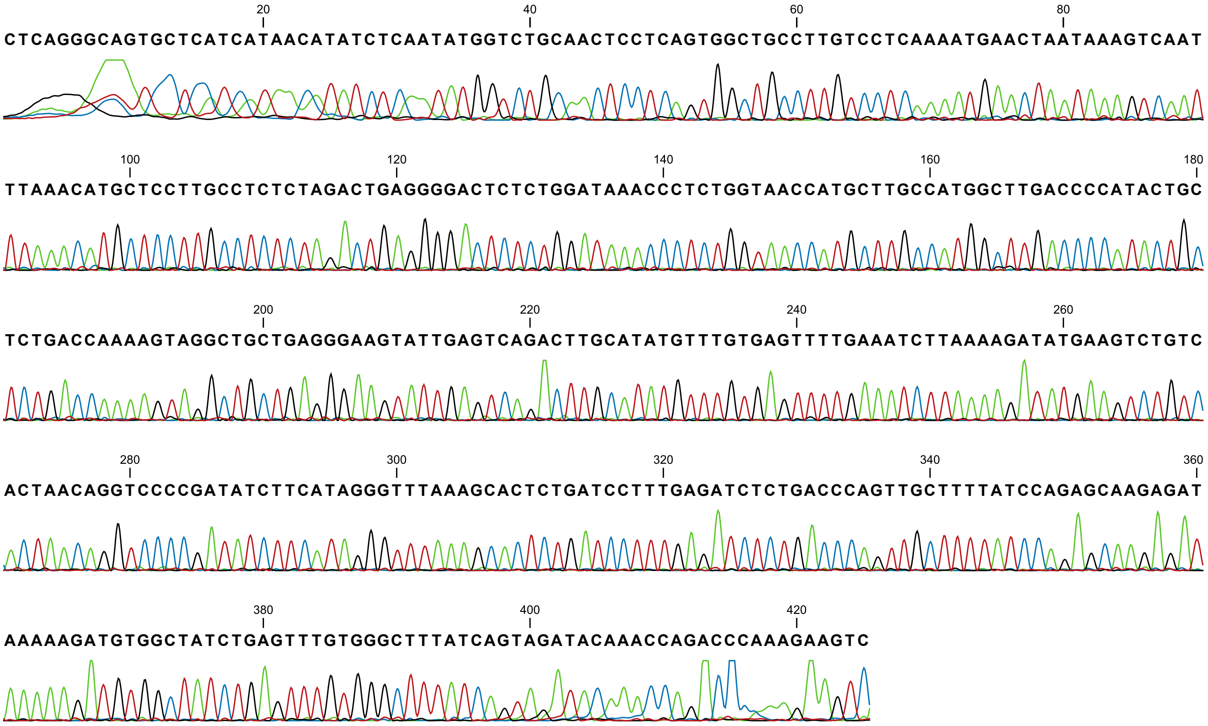

C6, Pangolin protoparvovirus (448 bp)

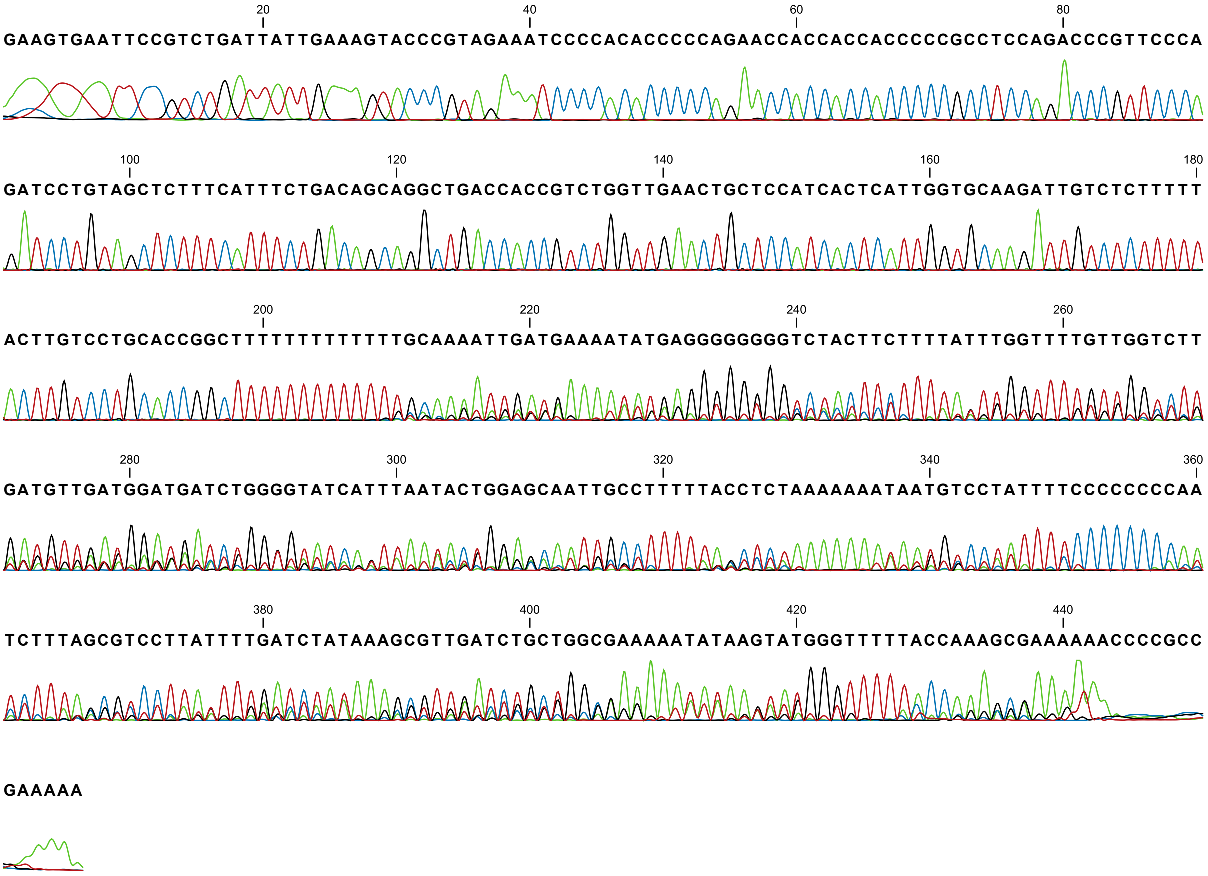

C7, Pangolin respirovirus (380 bp)

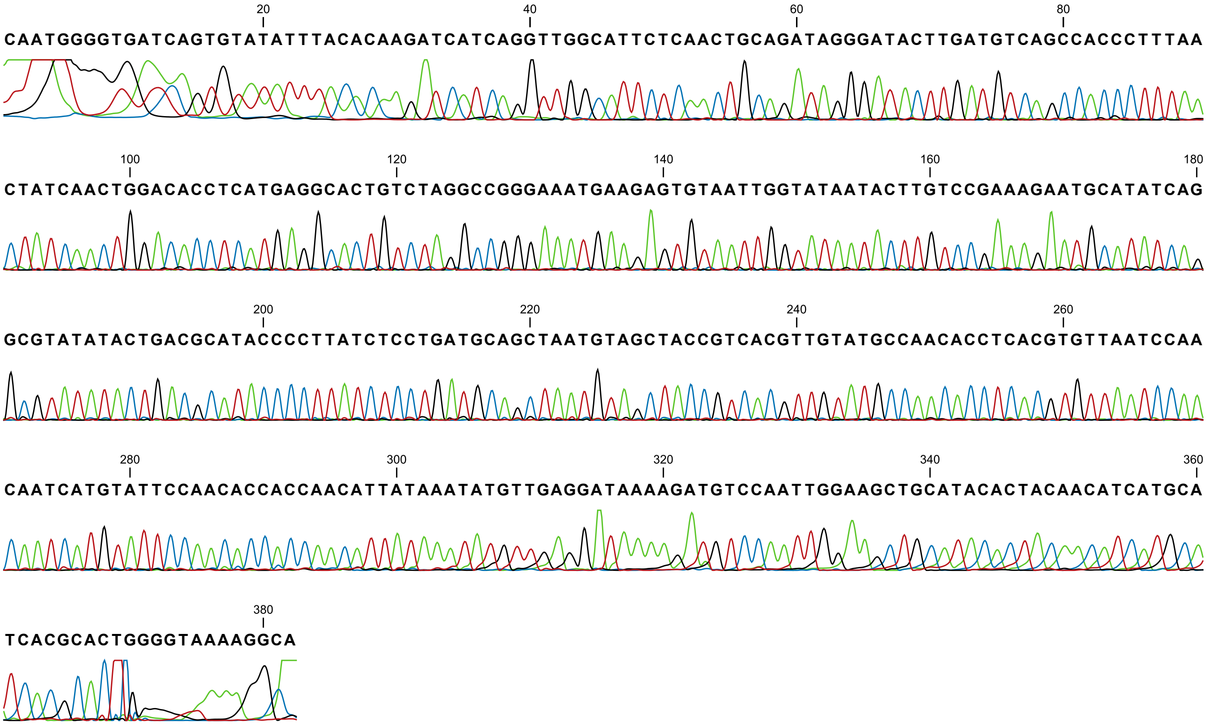

C9, Pangolin respirovirus (380 bp)

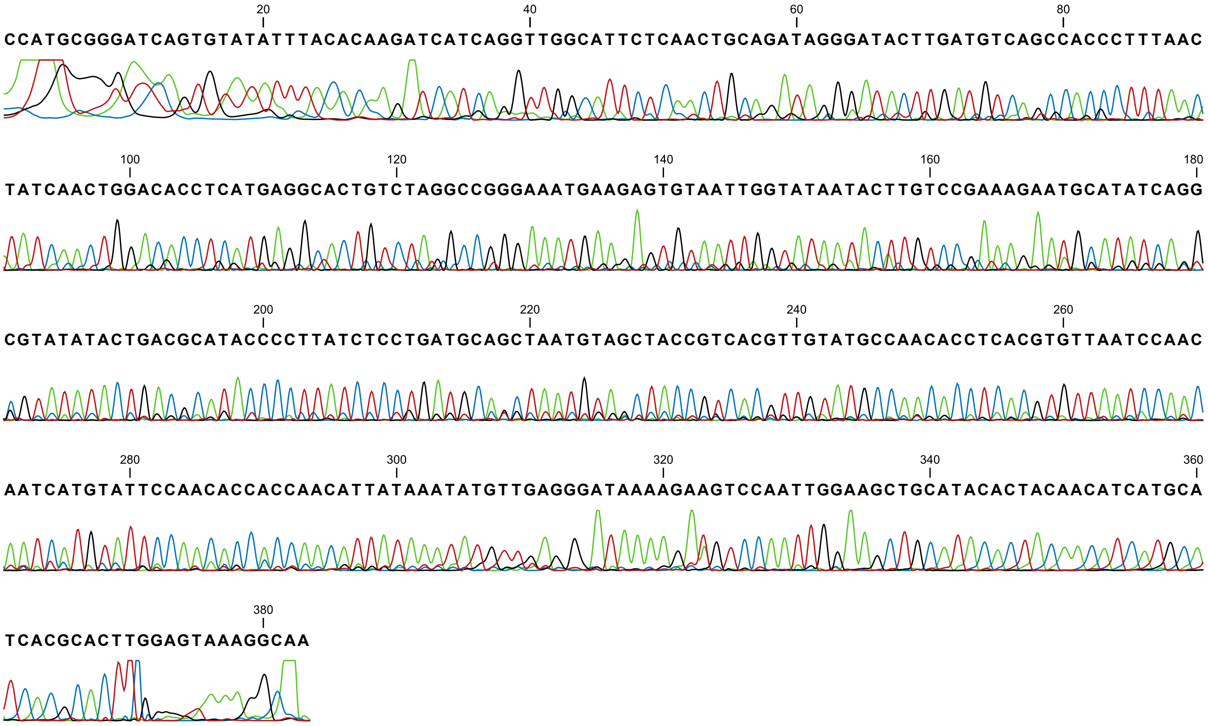

C10, Pangolin respirovirus (380 bp)

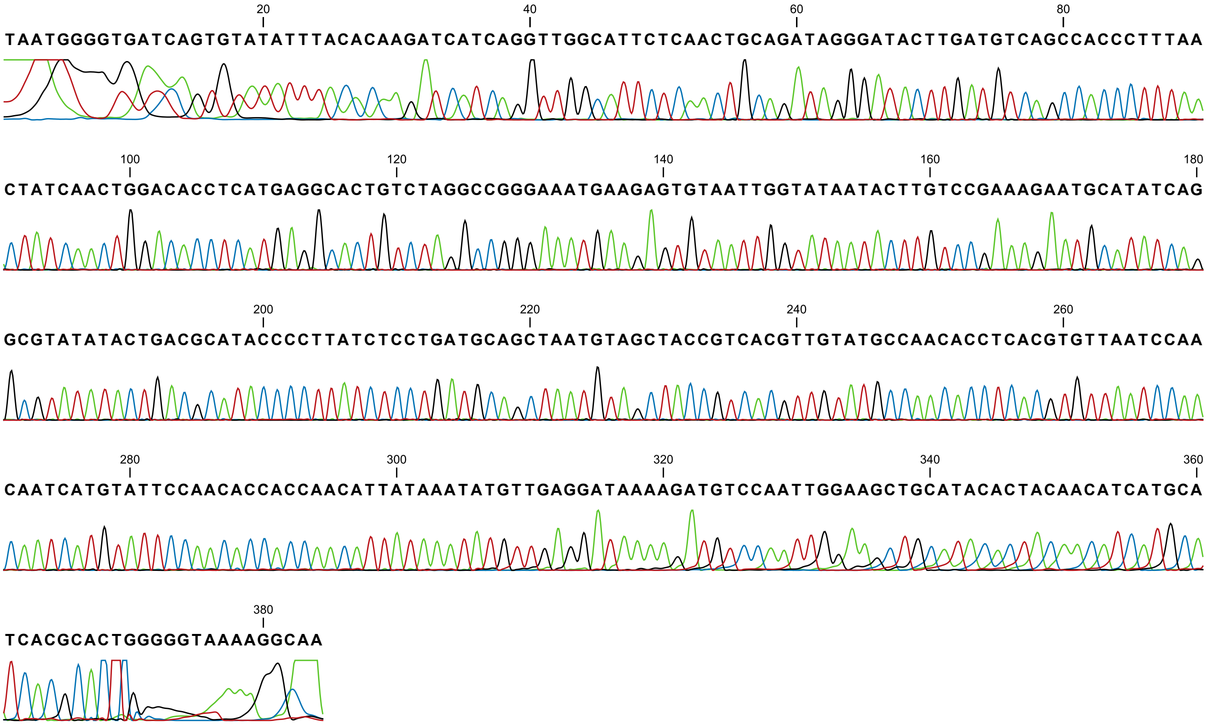

D1, Orthopneumovirus BIME1 (236 bp)

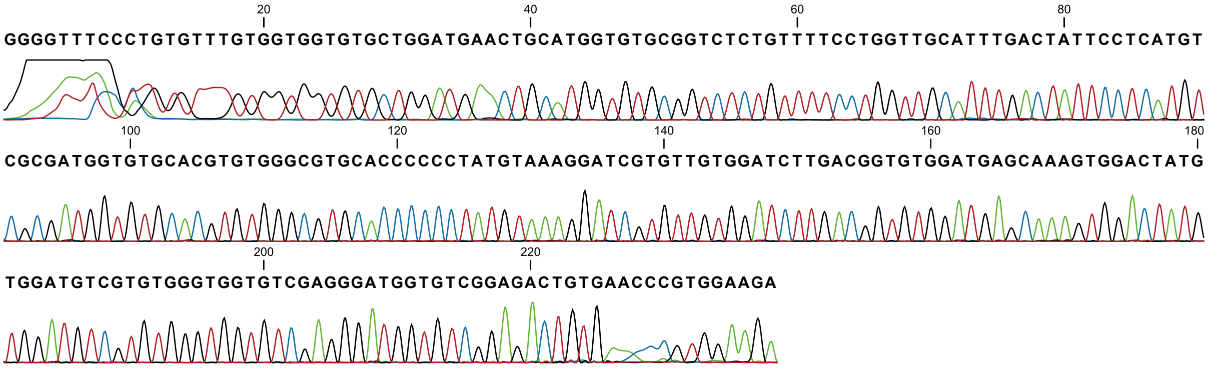

D7, Pangolin rotavirus A (227 bp)

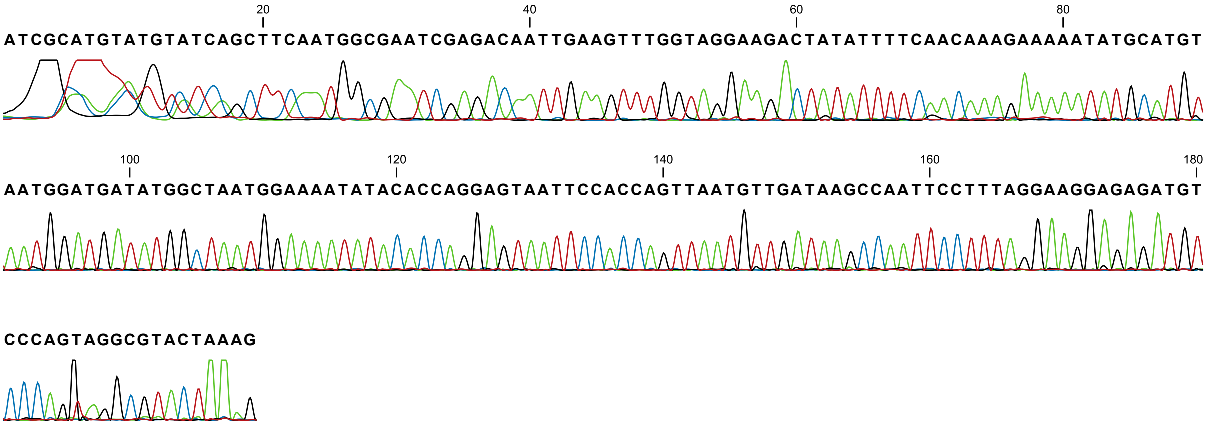

D8, Pangolin rotavirus A (227 bp)

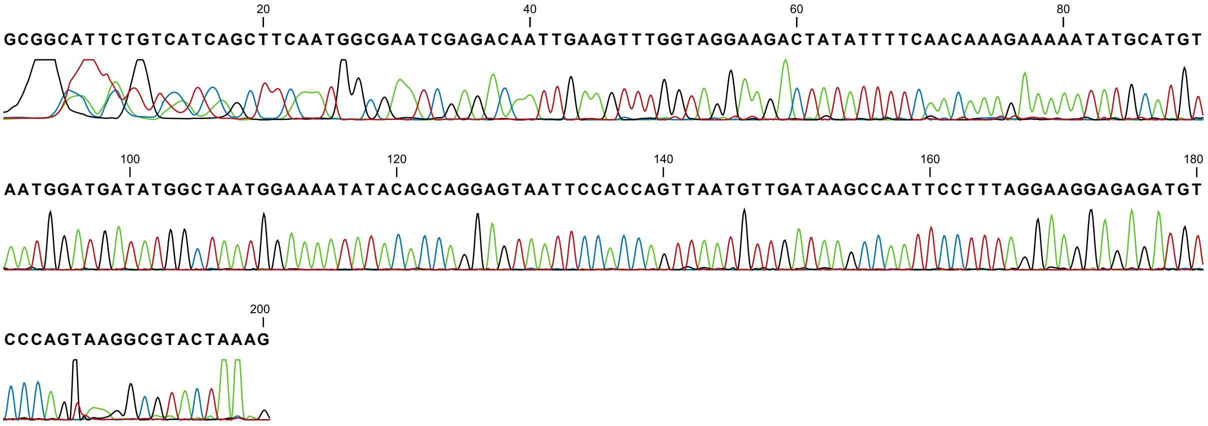

D9, Pangolin rotavirus A (227 bp)

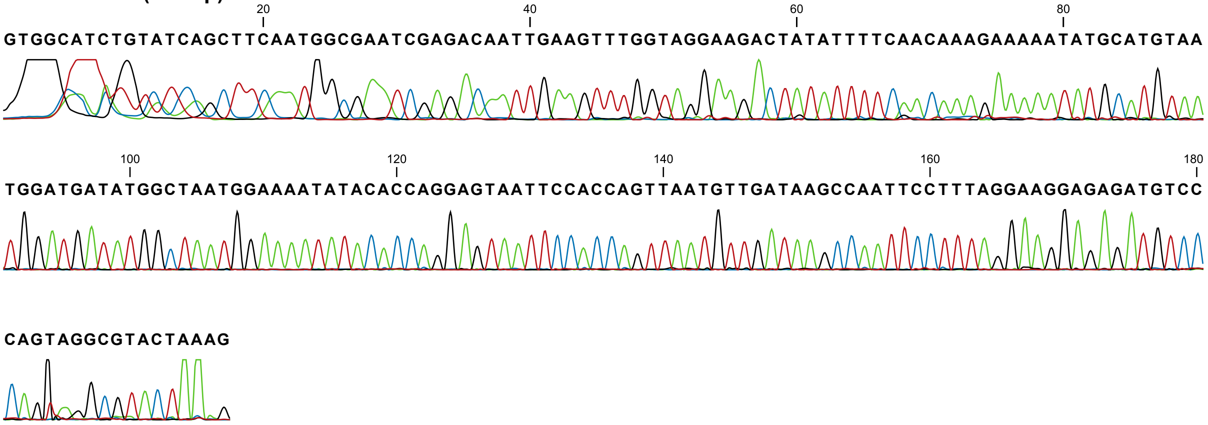

E1, Pangolin hunnivirus BIME2 (255 bp)

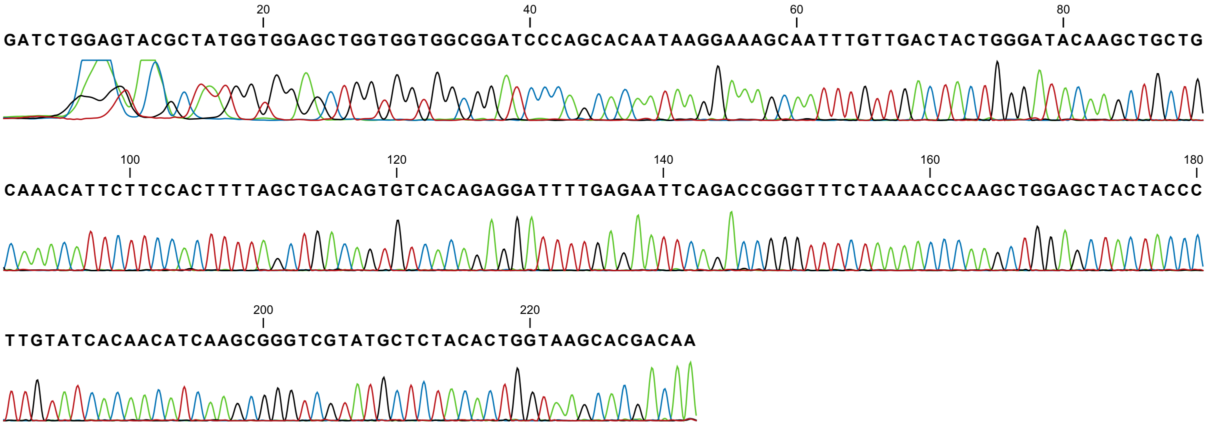

E2, Pangolin hunnivirus BIME2 (255 bp)

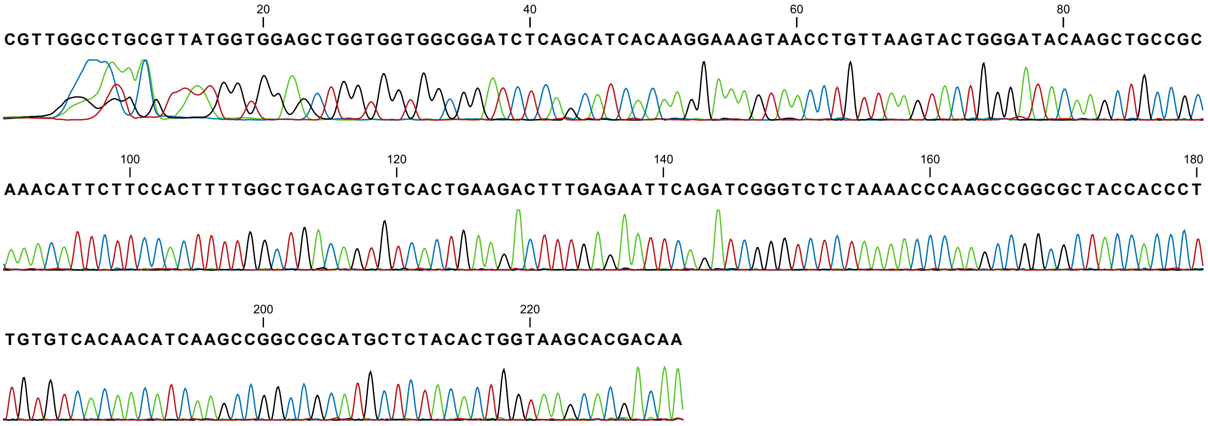

E3, Pangolin hunnivirus BIME2 (255 bp)

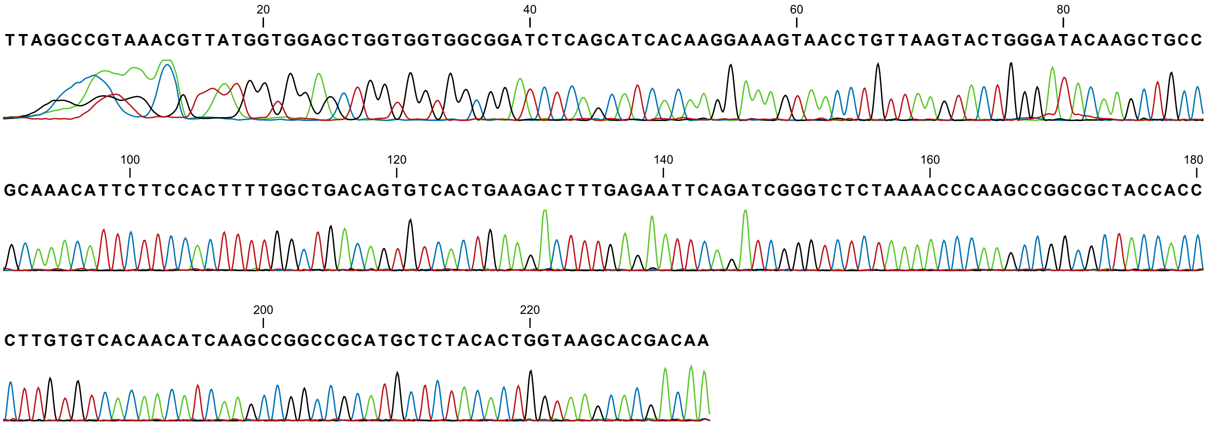

E7, Pangolin hunnivirus BIME3 (310 bp)

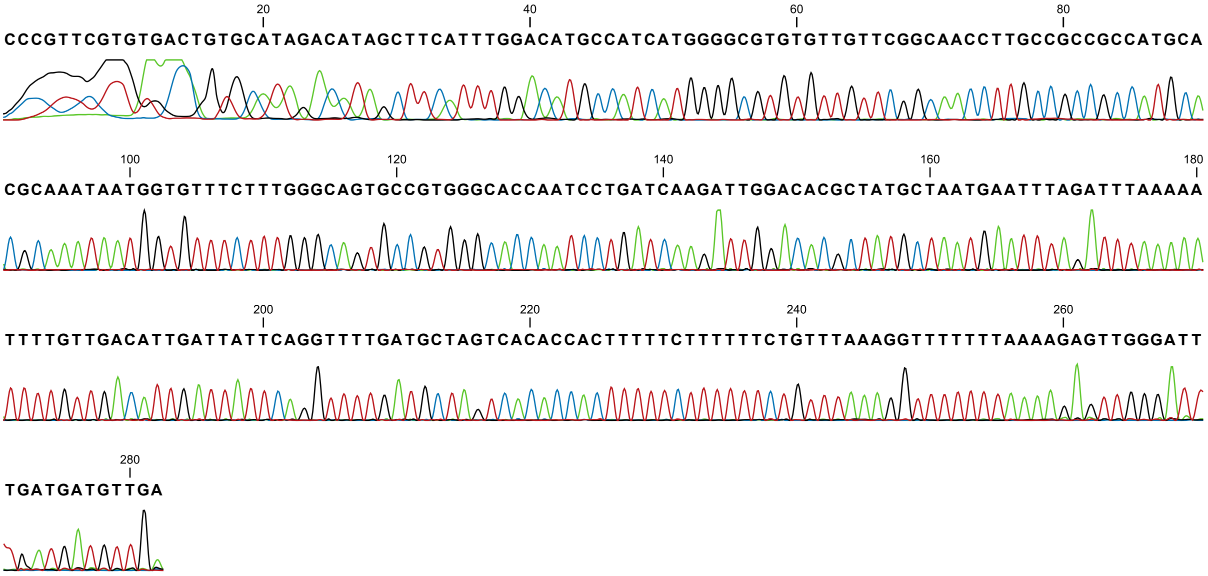

E9, Pangolin orthoreovirus (352bp)

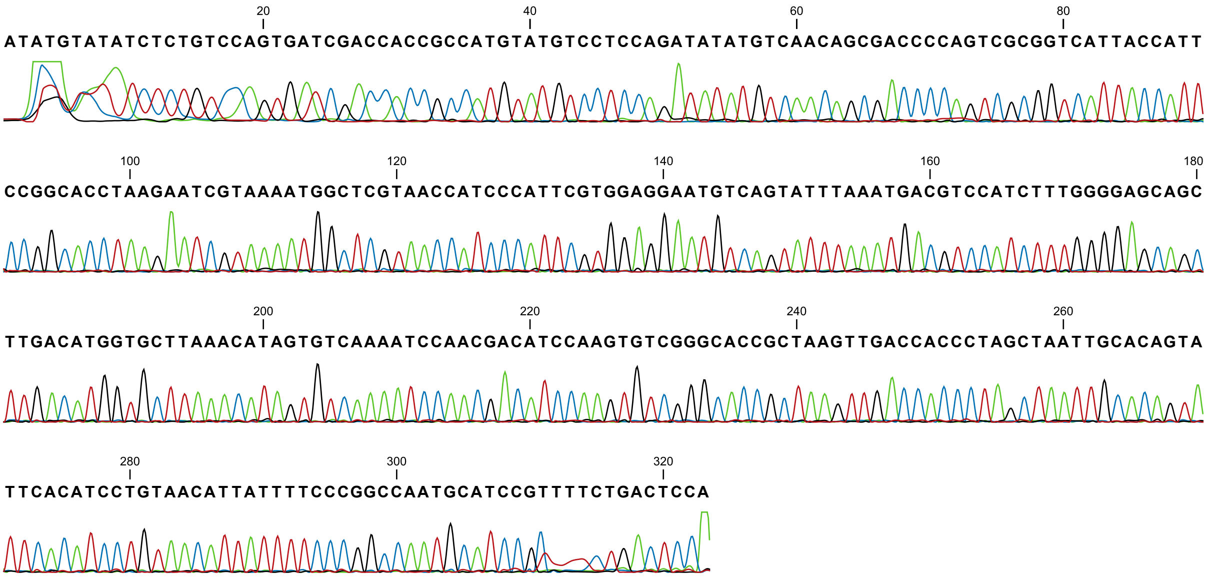

F2, Pangolin coronavirus HKU4 (275bp)

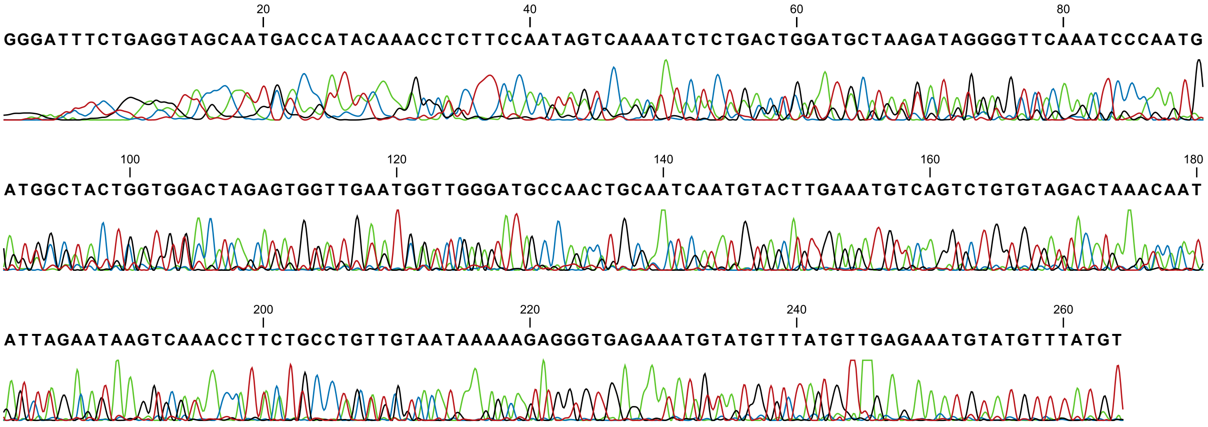

F3, Pangolin coronavirus HKU4 (275bp)

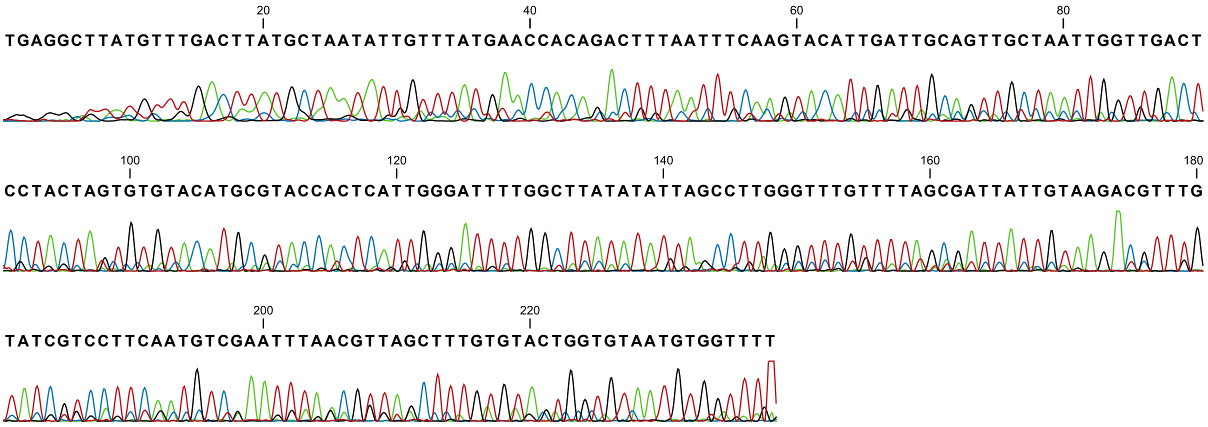

F5, Pangolin shanbavirus BIME1 (451bp)

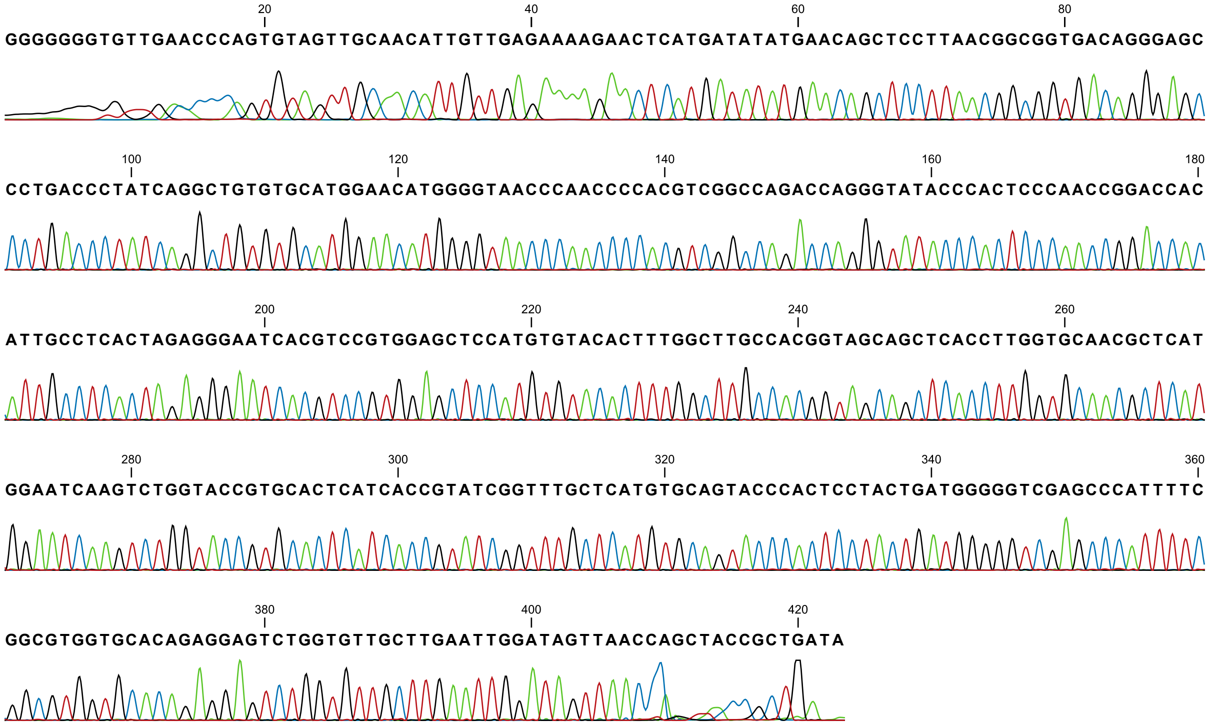

F6, Pangolin shanbavirus BIME1 (451bp)

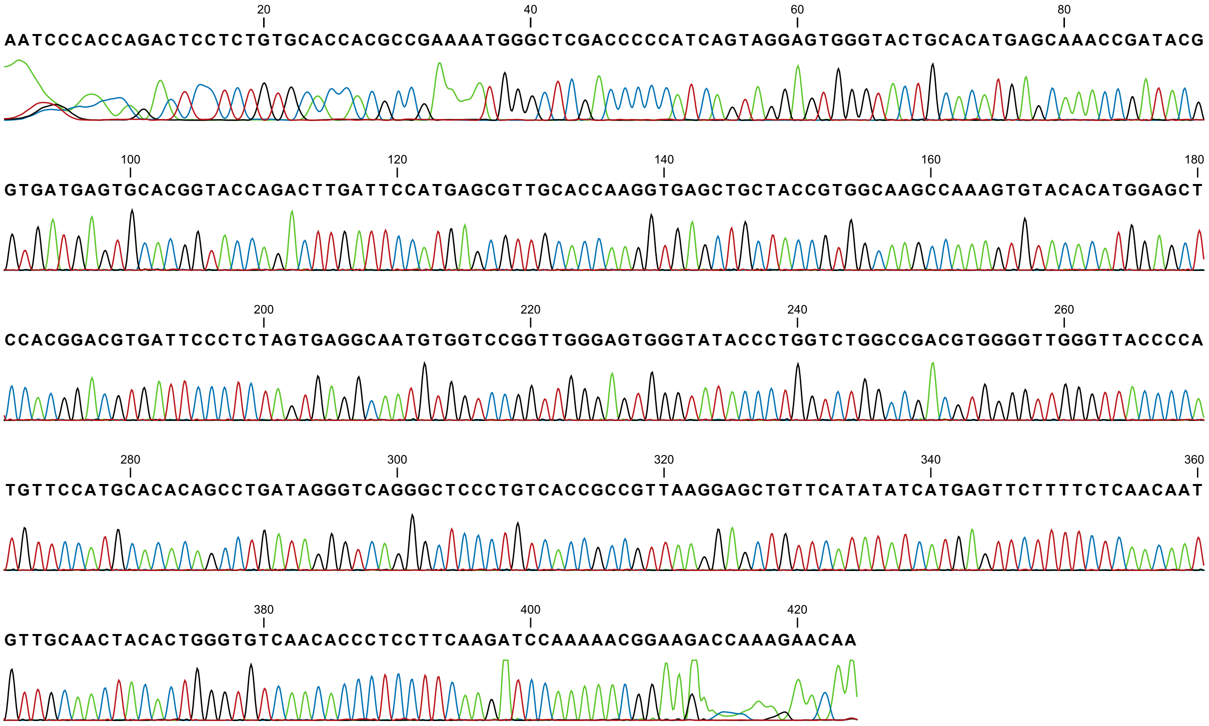

F7, Pangolin shanbavirus BIME1 (451bp)

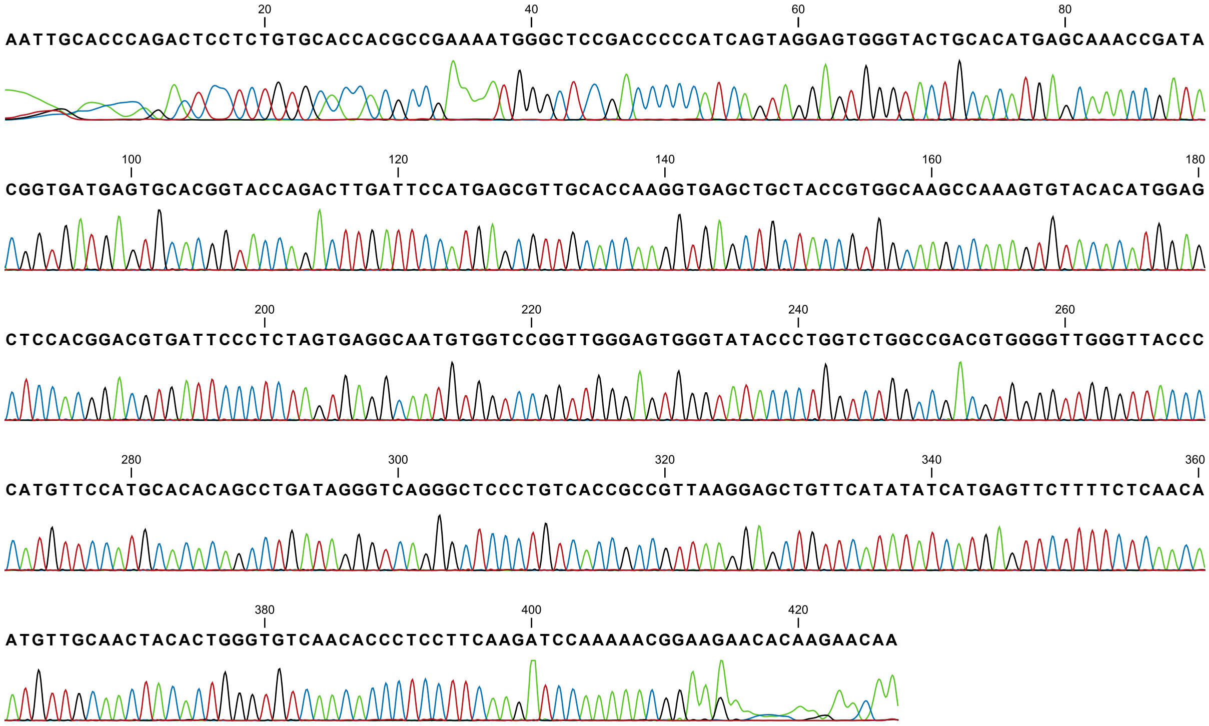

Supplementary Fig. 1. The diagrams of RT-PCT product sequenced by the Sanger sequencing.

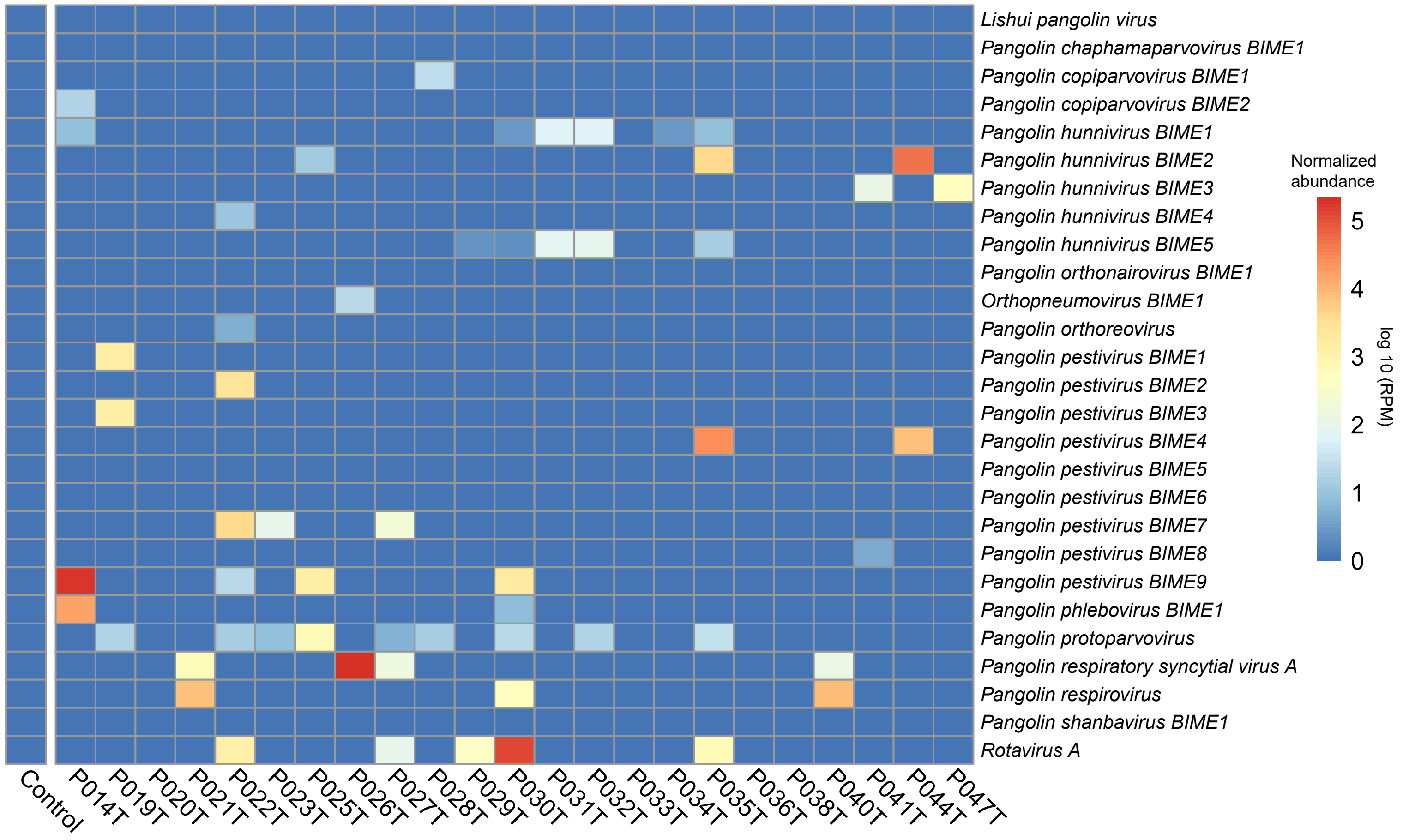

Supplementary Fig. 2. Viral abundance of the water control and the pangolin samples sequenced on the same run. Each cell in the heatmap represents the normalized number of reads of the given virus in the control and each sample, with an index-hopping threshold of 0.1%.
