# Supplementary material for: Trafficked Malayan pangolins contain viral pathogens of humans
Source: Nat Microbiol. 2022 Aug 2;7(8):1259–69. doi: 10.1038/s41564-022-01181-1 (PMC9352580; doi:10.1038/s41564-022-01181-1)
Supplement: Supplementary file 2 — Reporting Summary [file 41564_2022_1181_MOESM2_ESM.pdf]

nature portfolio

Corresponding author(s): Jia-Fu Jiang, Yan-Ling Hu and Wu-Chun Cao

Last updated by author(s): Jun 6, 2022

Reporting Summary

Nature Portfolio wishes to improve the reproducibility of the work that we publish. This form provides structure for consistency and transparency in reporting. For further information on Nature Portfolio policies, see our Editorial Policies and the Editorial Policy Checklist.

Statistics

For all statistical analyses, confirm that the following items are present in the figure legend, table legend, main text, or Methods section.

n/a	Confirmed	
☐	☒	The exact sample size (*n*) for each experimental group/condition, given as a discrete number and unit of measurement
☒	☐	A statement on whether measurements were taken from distinct samples or whether the same sample was measured repeatedly
☐	☒	The statistical test(s) used AND whether they are one- or two-sided<br>*Only common tests should be described solely by name; describe more complex techniques in the Methods section.*
☒	☐	A description of all covariates tested
☒	☐	A description of any assumptions or corrections, such as tests of normality and adjustment for multiple comparisons
☒	☐	A full description of the statistical parameters including central tendency (e.g. means) or other basic estimates (e.g. regression coefficient) AND variation (e.g. standard deviation) or associated estimates of uncertainty (e.g. confidence intervals)
☐	☒	For null hypothesis testing, the test statistic (e.g. *F*, *t*, *r*) with confidence intervals, effect sizes, degrees of freedom and *P* value noted<br>*Give P values as exact values whenever suitable.*
☒	☐	For Bayesian analysis, information on the choice of priors and Markov chain Monte Carlo settings
☒	☐	For hierarchical and complex designs, identification of the appropriate level for tests and full reporting of outcomes
☒	☐	Estimates of effect sizes (e.g. Cohen's *d*, Pearson's *r*), indicating how they were calculated

Our web collection on statistics for biologists contains articles on many of the points above.

Software and code

Policy information about availability of computer code

Data collection	Reference genome sequence data were downloaded from GenBank using the web interface.
Data analysis	Software used: Geneious Prime 2021.2.2, BLAST v2.3.0+, MEGAHIT v1.2.9, MAFFT v7.475, PhyML v3.1, IQTREE v1.6.10, fastp v0.21.0, DIAMOND v0.9.21, Bowtie2 v2.3.4.2, vegan v2.5-7, SimPlot v3.5.1, GATK v4.1.2.0, TrimAl v1.4.rev22, MrBayes v3.2.7

For manuscripts utilizing custom algorithms or software that are central to the research but not yet described in published literature, software must be made available to editors and reviewers. We strongly encourage code deposition in a community repository (e.g. GitHub). See the Nature Portfolio guidelines for submitting code & software for further information.

Data

Policy information about availability of data

All manuscripts must include a data availability statement. This statement should provide the following information, where applicable:

- Accession codes, unique identifiers, or web links for publicly available datasets
- A description of any restrictions on data availability
- For clinical datasets or third party data, please ensure that the statement adheres to our policy

The sequencing reads of 161 pangolins after quality control have been deposited in the NCBI Sequence Read Archive database under the BioProject accession number PRJNA845961. Pangolin mitochondrial sequences and viral sequences assembled in this study were deposited in the GenBank (OM009282-OM009284,OM037454, ON024072-ON024140, ON059801-ON059909, ON166559-ON166563, ON045154-ON045314). We have provided the GenBank accession numbers of each reference sequence in phylogenetic trees files (newick format) and associated sequence alignments (fasta format) available at https://doi.org/10.6084/m9.figshare.19499030.

Field-specific reporting

Please select the one below that is the best fit for your research. If you are not sure, read the appropriate sections before making your selection.

☒ Life sciences ☐ Behavioural & social sciences ☐ Ecological, evolutionary & environmental sciences

For a reference copy of the document with all sections, see nature.com/documents/nr-reporting-summary-flat.pdf

Life sciences study design

All studies must disclose on these points even when the disclosure is negative.

Sample size	No sample size calculation was performed. We collected available archived samples of pangolins smuggled from Southeast Asia to China and confiscated by Customs during 2018-2019. Available tissues (including muscle, lung, intestine, spleen, liver, heart and kidney) of each pangolin were pooled into a single sample. Libraries of 161 pangolin samples were successfully constructed for meta-transcriptome sequencing.
Data exclusions	No data were excluded.
Replication	We designed specific primers according to assembled virus sequences (Extended Data Fig. 1), and performed RT-PCR to confirm the presence of viruses identified by MGISEQ-2000 sequencing platform (Supplementary Table 1) followed by Sanger sequencing (Supplementary Fig. 1). All the PCR experiments were successful. Viruses identified by NovaSeq 6000 platform were not included in RT-PCR due to lack of sufficient original tissue samples.
Randomization	There was no separation of experimental groups in the study, hence no randomization.
Blinding	There was no separation of experimental groups in the study, hence no blinding.

Reporting for specific materials, systems and methods

We require information from authors about some types of materials, experimental systems and methods used in many studies. Here, indicate whether each material, system or method listed is relevant to your study. If you are not sure if a list item applies to your research, read the appropriate section before selecting a response.

Materials & experimental systems

n/a	Involved in the study	
☒	☐	Antibodies
☒	☐	Eukaryotic cell lines
☒	☐	Palaeontology and archaeology
☒	☐	Animals and other organisms
☒	☐	Human research participants
☒	☐	Clinical data
☒	☐	Dual use research of concern

Methods

n/a	Involved in the study	
☒	☐	ChIP-seq
☒	☐	Flow cytometry
☒	☐	MRI-based neuroimaging
