# Supplementary material for: Trafficked Malayan pangolins contain viral pathogens of humans
Source: Nat Microbiol. 2022 Aug 2;7(8):1259–69. doi: 10.1038/s41564-022-01181-1 (PMC9352580; doi:10.1038/s41564-022-01181-1)
Supplement: Source Data Extended Data Fig. 2 — Images. [file 41564_2022_1181_MOESM10_ESM.pdf]

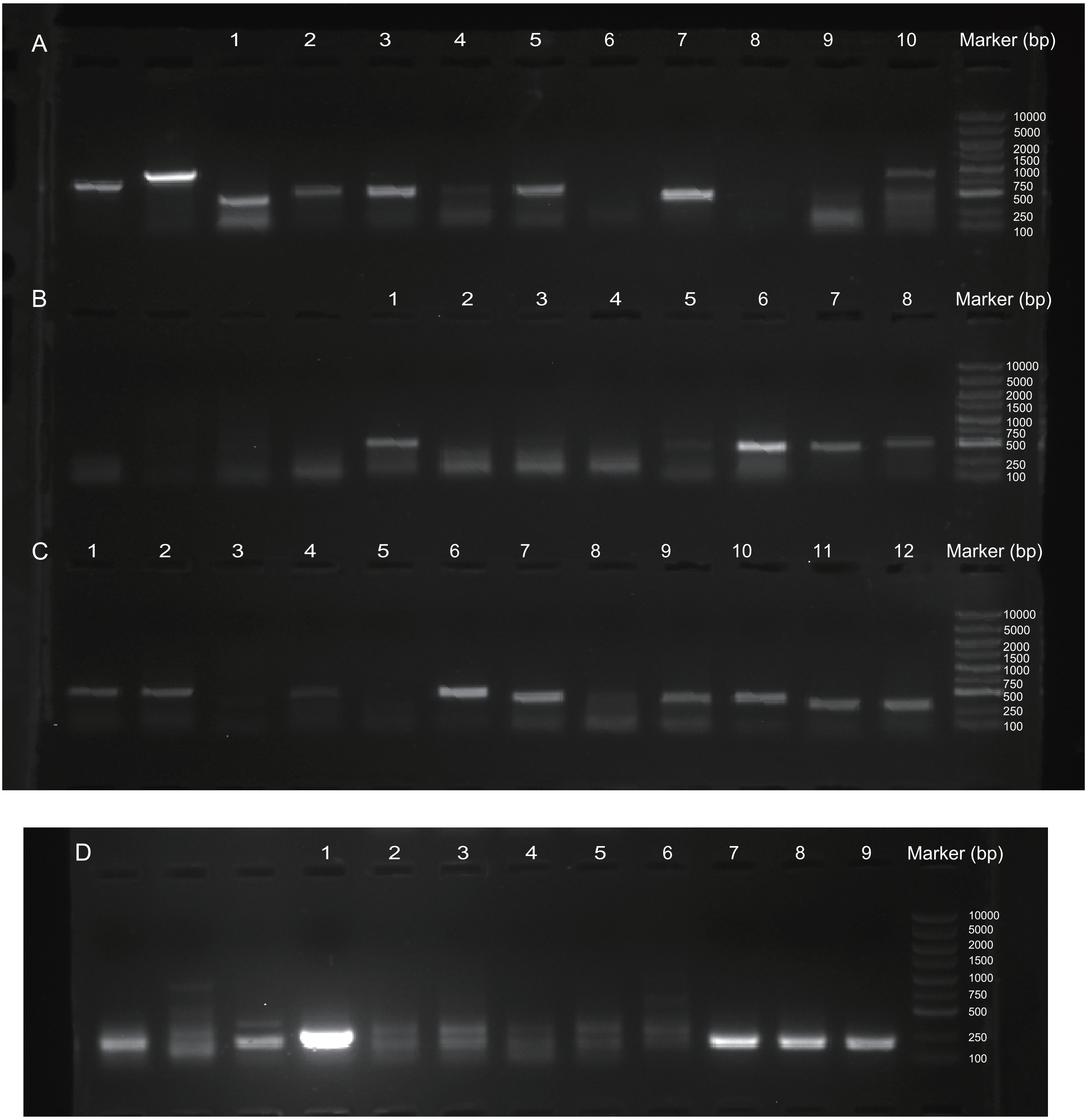

A1, Pangolin copiparvovirus BIME1 (330 bp)
A2, Pangolin RSV-A (450 bp)
A3, Pangolin RSV-A (450 bp)
A4, Pangolin hunnivirus BIME4 (481 bp)
A5, Pangolin hunnivirus BIME1 (467 bp)
A7, Pangolin hunnivirus BIME5 (410 bp)

B1, Pangolin pestivirus BIME9 (451 bp)
B5, Pangolin pestivirus BIME1 (407 bp)
B6, Pangolin pestivirus BIME3 (394 bp)
B7, Pangolin pestivirus BIME2 (397 bp)
B8, Pangolin pestivirus BIME7 (461 bp)

C1, Pangolin pestivirus BIME7 (461 bp)
C2, Pangolin pestivirus BIME7 (461 bp)
C4, Pangolin phlebovirus BIME1 (402 bp)
C6, Pangolin protoparvovirus (448 bp)
C7, Pangolin respirovirus (380 bp)
C9, Pangolin respirovirus (380 bp)
C10, Pangolin respirovirus (380 bp)

D1, Orthopneumovirus BIME1 (236 bp)
D7, Pangolin rotavirus A (227 bp)
D8, Pangolin rotavirus A (227 bp)
D9, Pangolin rotavirus A (227 bp)

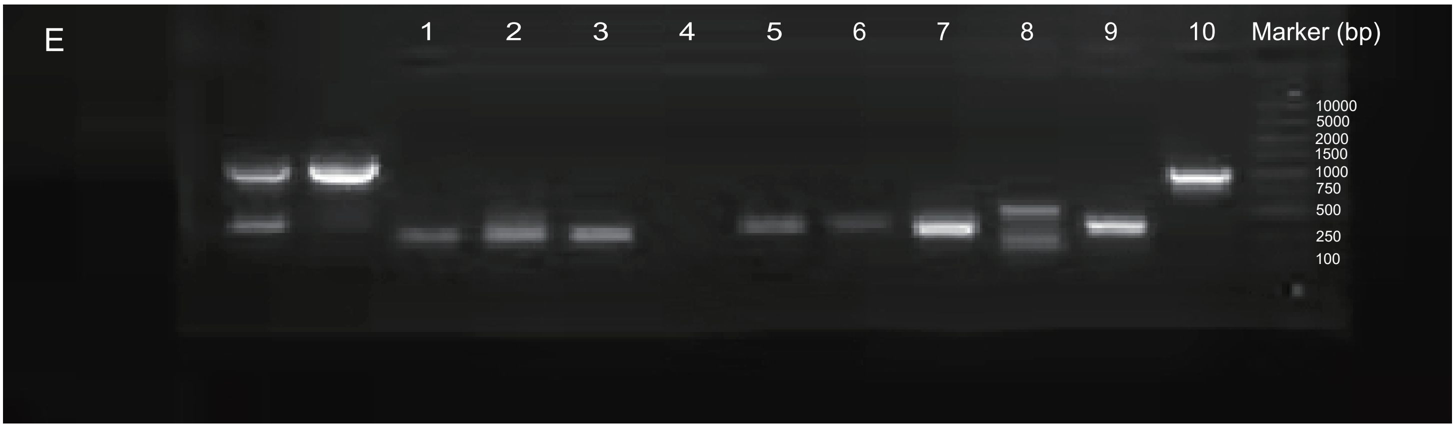

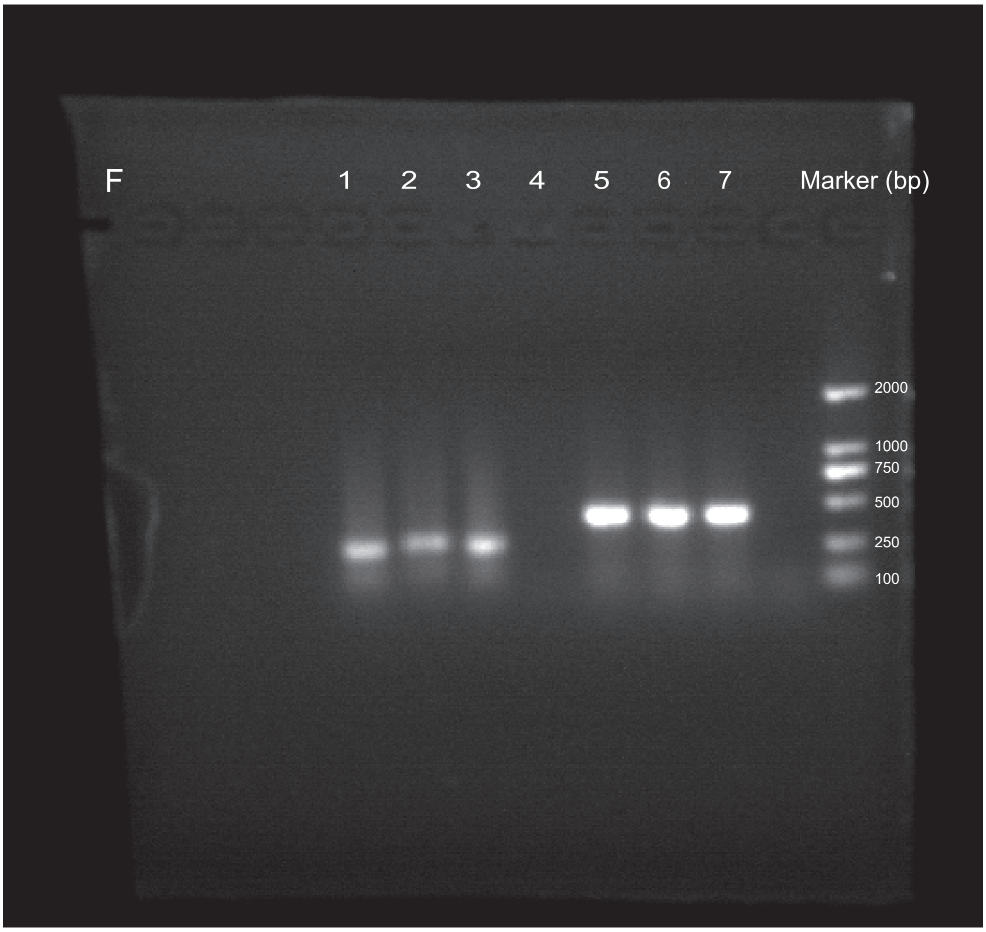

E1, Pangolin hunnivirus BIME2 (255 bp)
E2, Pangolin hunnivirus BIME2 (255 bp)
E3, Pangolin hunnivirus BIME2 (255 bp)
E7, Pangolin hunnivirus BIME3 (310 bp)
E9, Pangolin orthoreovirus (352 bp)

F1, Pangolin coronavirus HKU4 (275 bp)
F2, Pangolin coronavirus HKU4 (275 bp)
F3, Pangolin coronavirus HKU4 (275 bp)
F4, ddH_2O
F5, Pangolin shanbavirus BIME1 (451 bp)
F6, Pangolin shanbavirus BIME1 (451 bp)
F7, Pangolin shanbavirus BIME1 (451 bp)
